# Longitudinal multi-functional analysis identified responses of T cells, B cells, and monocytes as hallmarks of immunotherapy tolerance in patients with merkel cell carcinoma

**DOI:** 10.1371/journal.pone.0293922

**Published:** 2023-11-20

**Authors:** Quyuan Tao, Jia-xin Du, Shijing Zhang, Wenjia Lin, Yongxin Luo, Ying Liu, Jingyan Zeng, Xin-lin Chen

**Affiliations:** 1 School of Basic Medical Science, Guangzhou University of Chinese Medicine, Guangzhou, Guangdong, China; 2 Shenzhen Clinical College, Guangzhou University of Chinese Medicine, Guangzhou, China; Korea Disease Control and Prevention Agency, REPUBLIC OF KOREA

## Abstract

**Purpose:**

Merkel cell carcinoma (MCC) is a neuroendocrine carcinoma originating in the skin. Studies are needed to determine the mechanisms of immune escape in patients with MCC, and malignant cell conditions that promote immune evasion.

**Methods:**

We used Single-cell RNA sequencing (scRNA-seq) to determine cellular features associated with MCC disease trajectory. A longitudinal multi-omics study was performed using scRNA-seq data of peripheral blood harvested from four-time points. Six major cell types and fifteen cell subgroups were identified and confirmed their presence by expression of characteristic markers. The expression patterns and specific changes of different cells at different time points were investigated. Subsequently, bulk RNA data was used to validate key findings.

**Results:**

The dynamic characteristics of the cells were identified during the critical period between benign improvement and acquisition of resistance. Combined with the results of the validation cohort, the resistance program expressed in the relapse stage is mainly associated with T cell exhaustion and immune cell crosstalk disorder. Coinciding with immune escape, we also identified a decrease non-classical monocytes and an expansion of classical monocytes with features of high inflammation and immune deficiency.

**Conclusion:**

Changes in cellular status, such as depletion of T cells and dysregulation of B cell proliferation and differentiation, may lead to drug resistance in MCC patients. Meanwhile, the widespread decreased antigen presentation ability and immune disorders caused by deletion of MHC class II gene expression should not be ignored.

## Introduction

Merkel cell carcinoma (MCC) is a rare and aggressive neuroendocrine carcinoma originating in the skin, usually caused by Merkel cell polyomavirus (MCPyV) [1–3]. Due to the characteristics of high misdiagnosis rate, poor prognosis, easy local recurrence, and metastasis [4], MCC has a 5-year survival rate of 40% and a fatality rate of 33%, which is even higher than melanoma [1]. Standard MCC therapies are mainly divided into the following two types: Primary and metastatic lymph node tumors are treated with surgery and radiotherapy as the mainstay. Unresectable and metastatic tumors were treated with cytotoxic chemotherapy and immune checkpoint inhibitors (ICIs) as the mainstay [5]. Nghiemet et al. reported a 56% response rate in patients with advanced MCC, receiving anti-*PD1* antibody pembrolizumab to block the immune checkpoint [6]. The 6-month progression-free survival rate was 67% for immunotherapy, compared with 24% for chemotherapy [7,8]. These studies showed that immunotherapy had good curative effect and great potential for patients with advanced MCC. However, drug tolerance and immune escape associated with immunotherapy have become key issues in the clinical use of ICIs.

ICIs have changed the therapeutic landscape of several types of cancer [9], especially MCC [10]. Nonetheless, many patients with MCC acquired resistance after immunotherapy, which is often intrinsic [11–13]. The mechanism of ICIs resistance in MCC was unknown. ICIs target cell-cell interactions, so resistance may derive from different cells and their interactions in the tumor ecological system. Among several possible mechanisms for the acquired resistance, immune damage caused by loss of expression of major histocompatibility complex (MHC) molecules was the most prevalent [14–16]. T cell infiltration level was also thought to be associated with acquired resistance [17], but the complex mechanisms involved in drug resistance obviously more than that. Among many factors, the biological relationship between cell subsets and different temporal pathological stages may be important for us to elucidate the mechanisms of drug resistance and recurrence.

Compared with conventional bulk sequencing, single-cell RNA sequencing (scRNA-seq) is more outstanding in profiling cancer immune interactions and producing reliable response biomarkers [18]. In this study, different cell clusters in the peripheral blood mononuclear cells (PBMCs) of a patient with drug-resistant MCC were identified. We explored the proliferation potential and genetic relationships of T cells at the levels of differentiation and evolution. By studying the expression patterns of genes in PBMCs, we found a series of highly expressed genes associated with drug resistance and immune avoidance pathways. Meanwhile, these genes may serve as cellular markers. Moreover, we further revealed the evolutionary routes of various cellular from recovery to relapse in the same patient. Dissecting the immune checkpoint dysregulation and immune escape mechanisms occurring in the immunotherapy of this patient, is beneficial for understanding the reasons behind the immunotherapy failure and cancer recurrence in MCC. This, in turn, further promotes the development of targeted treatment approaches for MCC.

## Materials and methods

### Single-cell RNA-seq data acquisition and processing

ScRNA-seq data (GSE117988) has been described by Paulson KG et al [19]. Patients with advanced MCC were treated with autologous ex vivo expanded CD8+ T cells, and experienced an initial reduction in overall tumor pressure but eventually relapsed. Drug resistance occurred during the 22-month clinical response period, and PBMCs samples were collected at four time points for 10X single-cell sequencing: pre-treatment (Pre), early post treatment day + 27 (D+27), responding post treatment day + 376 (RespD376), relapse/acquired resistance post treatment day + 614 (ARD614).

We performed simple filtering of the expression matrix by quality control, dimension reduction, and feature selection with the Seurat package (R version 4.0.2) [20]. Cells expressing more than 500 genes and genes expressing more than 10% of the cells were selected for further analyses. Cells with detected gene numbers < 500 or > 6,000 and mitochondrial unique molecular identifiers (UMIs) > 10% were removed. Then the filtered expression matrix was normalized using NormalizeData function, and the top 2,000 genes with a high degree of intercellular variation were calculated using FindVariableFeatures function. RunPCA function was used to reduce the dimensionality of the data followed by nonlinear dimensional reduction.

### Cell type annotation and cluster marker identification

Dimensionality reduction was performed for the gene-barcode matrix using PCA and UMAP analyses. The first 10 principal components (PCs) were retained and selected for nonlinear dimensional reduction. These PCs were projected into two-dimensional space using UMAP. To cluster the cells, share-nearest neighbor (SNN) graph method was implemented in Seurat [20]. Subsequently, the distinct clusters of cells were divided according to a neighborhood size of 40 and a resolution parameter of 0.6. To optimize feature selection, three different cell clusters were excluded because of likely blood contamination. Cluster-specific markers were identified via differential expression using FindMarkers function in Seurat. Clusters were then annotated and classified on the basis of the expression of typical markers of specific cell types.

### Differential expression genes analysis

In order to further explore the mechanism of tumor recurrence, we conducted a longitudinal differential expression analysis of cells at RespD376 and ARD614 time points. Differentially expressed genes (DEGs) between the two above time points were identified by applying FindMarkers function in Seurat. DEGs in each cell type were identified with false discovery rate (FDR) adjusted *p*-value of less than 0.05.

### Co-expression analysis

To reflect the expression regulatory relationship among DEGs, the WGCNA package (version 1.70.3) was used to identify the co-expressed modules of DEGs. All the DEGs identified from the above longitudinal analysis (981 genes) were used to obtain the gene co-expression modules. First, we calculated pairwise gene correlations based on the log-transformed normalized expression counts matrix across all cells. A soft threshold function was used to construct a signed adjacency matrix, which was the basis for constructing the topology overlap matrix (TOM) for the next step. The TOM was used to construct a gene tree by hierarchical clustering. DEGs were then split into different modules according to the gene tree by using CutreeDynamic function, while the minimum of the module size was set to 15. Then mergeCloseModules function with a parameter cut height set to 0.45 was used to merge modules that were closely associated. Moreover, the module eigengene values for each time point were calculated.

### Cell-Cell communication analysis

The receptor-ligand interactions that exist in different cell types were confirmed based on the significant DEGs that have been identified in these cell clusters. The reference data set for these ligand-receptor pairs come from the R package iTALK [21]. If the average expression of a ligand or receptor in a certain cell type is above the threshold of 0.5 (*log*_2_ (*average expression*)>0.5) and the ligand or receptor is expressed more than 10% of the cells, the cell type is defined as "expressing" the ligand or receptor transcript. After the expression of the receptor or ligand is determined, the corresponding interaction will be defined as incoming or outgoing of that cell type, respectively.

### Subclustering of major interested cell types

For the cell types identified as important or of interest by the previous analysis, cells were first extracted from the integrated data list. Next, these major cell types were divided into further subclustering by linear and nonlinear dimensionality reduction methods.

### Exhaustion score calculation

In order to assess the level of exhaustion of cell subtypes and identify cell subtypes with exhaustion features, we utilize the AddModuleScore function in Seurat to perform a characteristic scoring of cells based on the expression of *TIGIT*, *PDCD1*, *CTLA4*, *CD27*, *LAG3*.

### Bulk RNA data validation

A total of 124 samples were included in the validation dataset. These samples were collected from GSE39612 cohort of GEO database, which included tumor tissue from 60 MCC patients and 64 normal tissues as controls. Deconvolution algorithm Cibersort was used to quantify tumor-infiltrating immune cells from second-generation sequencing data. The expression matrix of top 50 genes specifically expressed in each cell type was extracted as the reference data set according to the result of cell cluster annotation.

### Functional enrichment analysis

Gene set variation analysis (GSVA) was executed using R package GSVA (version 1.38.2) [22] to quantify the pathway enrichment score across time-points or cell types. Metabolic pathway activities were evaluated using a curated dataset that contained 85 metabolic pathways [23]. Limma R package (version 3.38.3) was used to calculate differential activities of pathways between time-points or cell types. Pathway with a corrected *P* ≤ 0.01 was identified significantly disturbed.

## Results

### MCC patients exhibit acquired resistance that depends upon changes in the distribution of cell clusters in different stages

**Fig 1:**
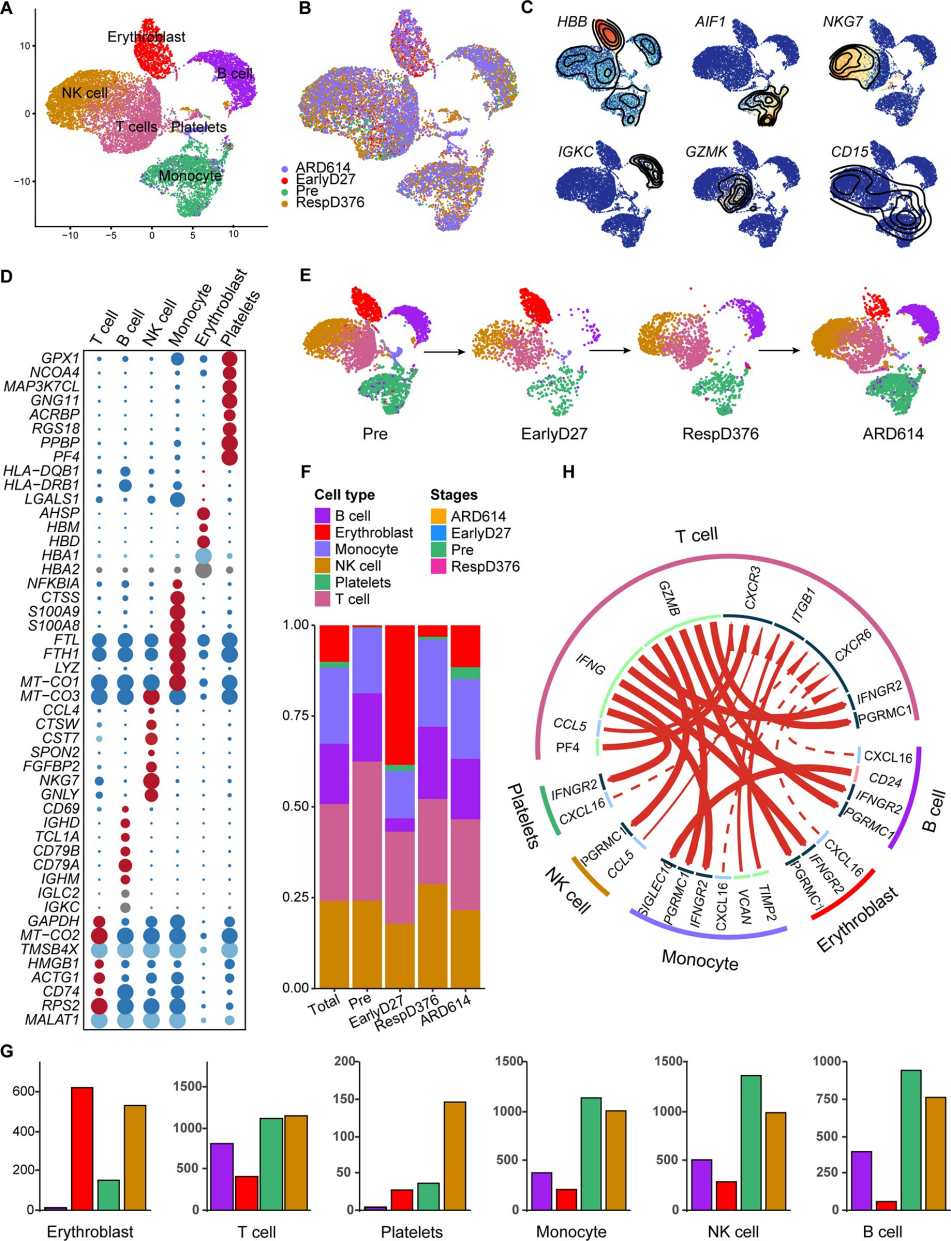
Overview of MCC at the cellular level. (A) Cellular populations identified. The UMAP projection of 12874 single cells from MCC samples shows the formation of 6 main clusters with label names. Each dot corresponds to a single cell, colored according to cell type. (B) Pseudotime UMAP representation of all merged cells, colored by time point. (C) Canonical cell markers were used to label clusters by cell identity as represented in the UMAP plot. (D) Dot plot for cell-type-specific signature genes. Color discriminates genes with increased (red) or decreased (blue) expression, and point size represents the number of cells per group expressing the corresponding gene. (E) UMAP plot of cluster breakdown by time point. (F) The average proportion of six main cells among different time points. (G) Percentages of the six types among four groups. Groups are shown in different colors. (H) Circos plot showing the interactions among different cell types in MCC.

We projected the MCC scRNA-seq data onto a UMAP figure and resolved the cell populations characterized by transcriptional signatures and stages of MCC patients respectively (Fig 1A and 1B). Six cell phenotypes were identified: T cell, B cell, NK cell, monocyte, erythroblast, and platelets. The expression of common markers in these cell types were shown in Fig 1C and 1D. Relative percentages of the six cell phenotypes were calculated relative to the stages of patients with MCC (Fig 1E and 1G). There was an increase in the percentage of lymphocytes, including T cell, B cell, and monocytes (Fig 1G). Cell-cell communication among six cell phenotypes were presented in the interaction plot (Fig 1H). The relationship between ligands and receptors was shown in the inner circle. T cells played an important role in cell-cell communication, followed by monocytes and B cells.

**Fig 2:**
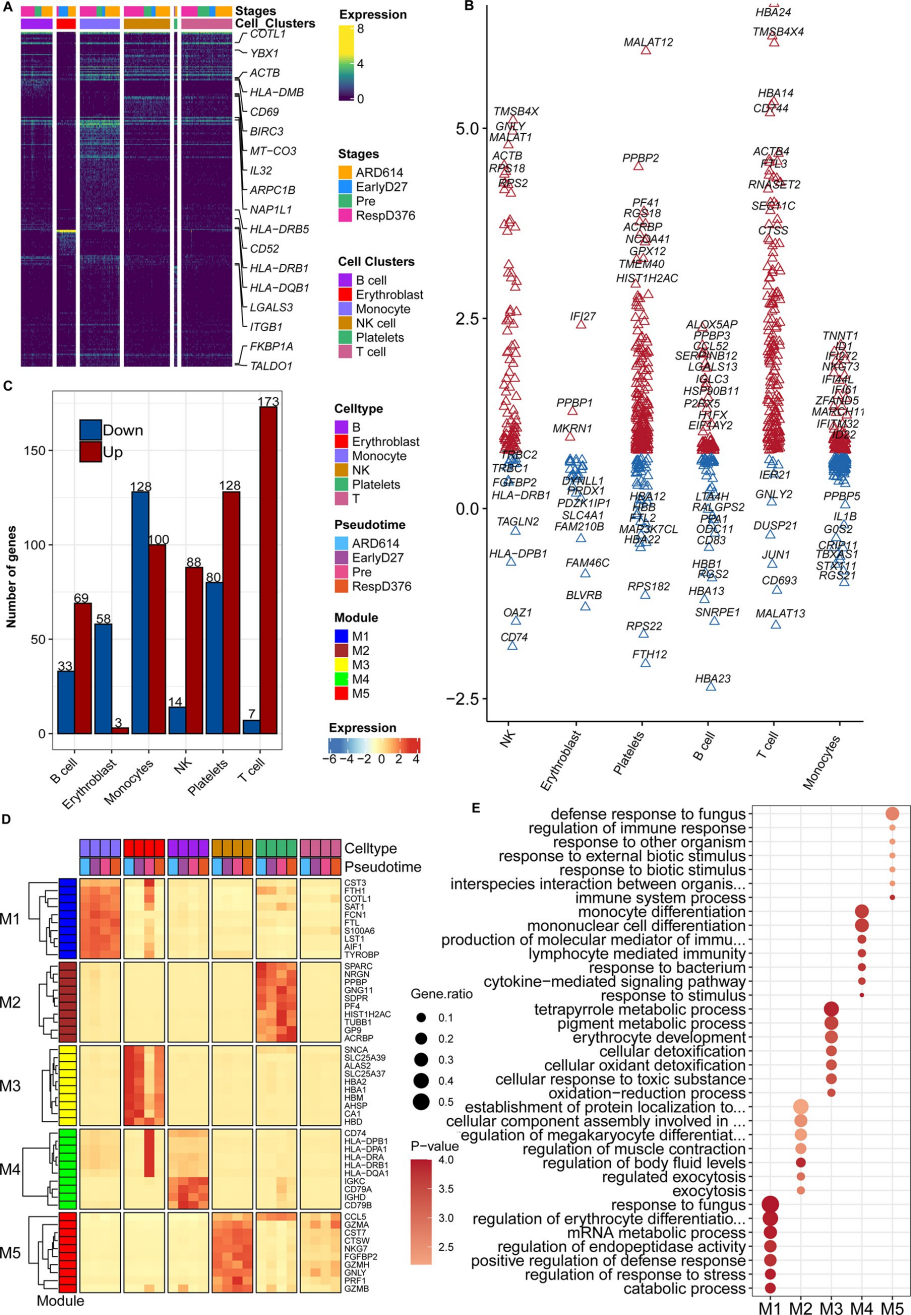
Co-expression analysis of differentially expressed genes. (A) Heatmap showing the expression of the top 18 differentially expressed genes for each cell cluster (*q* < 0.05). (B) Differential genes (DEGs) of six major cell types between groups RespD376 and ARD614. Colors discriminate DEGs with upregulation (red) or downregulation (blue) expression. The Y-axis represents the log2-fold change of DEGs. (C) Number of DEGs between RespD376 and ARD614 in each cell type. Colors discriminate upregulation (red) or downregulation (blue) expression. (D) Heatmap showed the average expression of module hub genes in different cell types and time points. (E) The dot plot showed the pathways enriched in specificity in the five modules.

The heatmap exhibited some of the genes characteristically expressed in different cell types at different times (Fig 2A). To capture the determinants that drive MCC from a benign response to resistance, DEGs in the stages of RespD376 and ARD614 were further analyzed (Fig 2B). In other words, we divided six kinds of cells into group RespD376 and group ARD614 for difference analysis. The number of DEGs obtained in each cell type were illustrated in Fig 2C (A total of 981 DEGs). Subsequently, 981 DEGs identified from RespD376 and ARD614 were used for weighted gene co-expression network analysis. Five modules were identified in total, which were defined as M1–M5, of co-expressed genes following specific expression patterns throughout the MCC disease phases (Fig 2D). For example, M4 module genes were mainly involved in monocyte differentiation, cytokine-mediated signaling pathway, and lymphocyte-mediated immune response (Fig 2E).

### A proliferative exhausted CD8 + T cell subpopulation emerges in the late relapse stage of MCC patient

**Fig 3:**
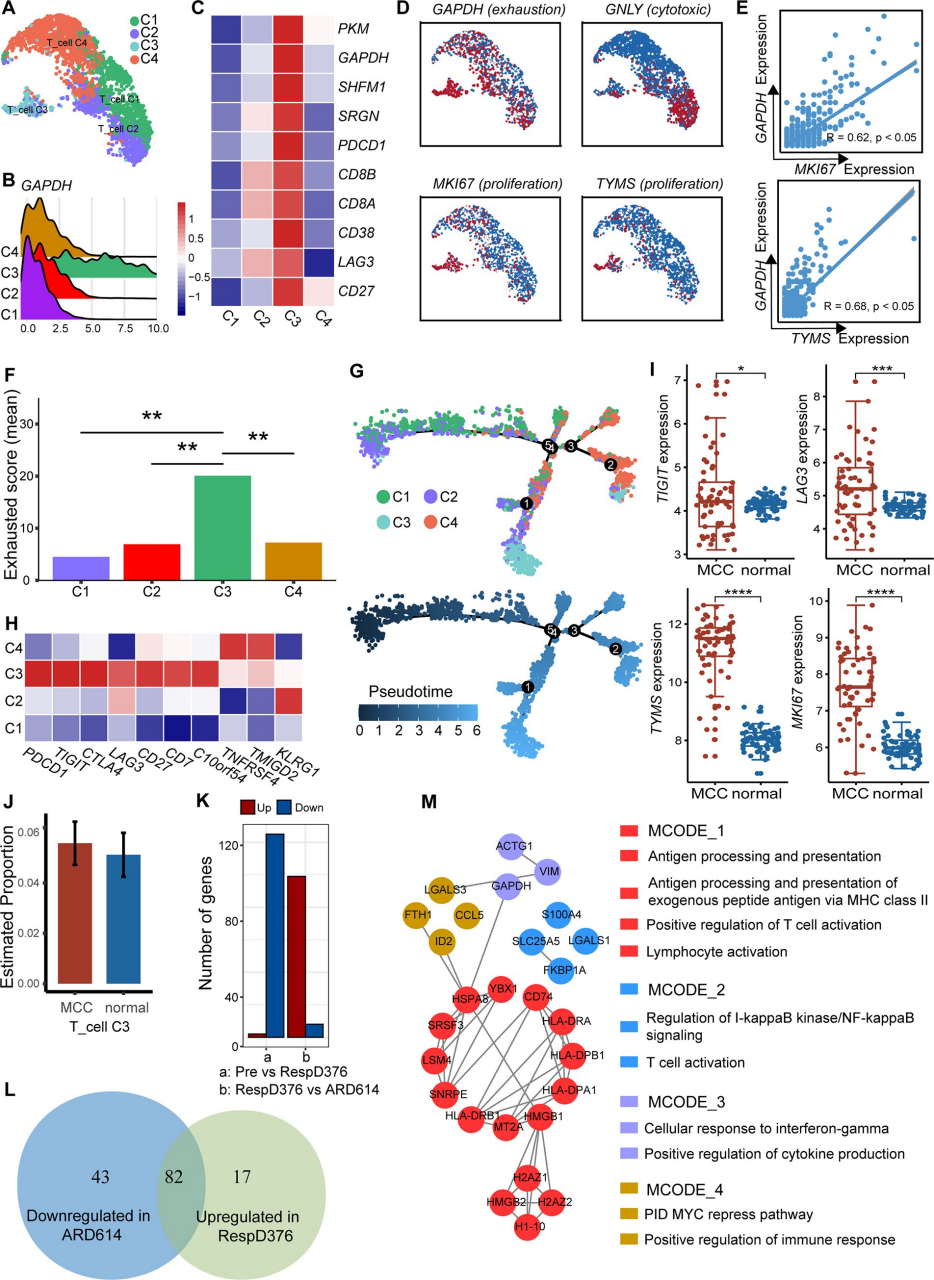
T cell changes across the MCC disease trajectory. (A) T cell compartment subtypes are represented as a UMAP. (B) Expression of GAPDH in four T cell subtypes. (C) Expression of the exhaustion-related markers. (D) Expression levels of various subtype markers in T cells. Red dots indicate high expression and blue dots indicate low expression. (E) Correlation between proliferation marker expression and *GAPDH* expression in C3 cluster cells. (F) Exhaustion score calculated based on expression levels of exhaustion markers. “**” indicates that the T-test *p*-value is less than 0.05. (G) Differentiation trajectory of *CD8*+ T cells in MCC, with each color-coded for clusters (top) and pseudotime (bottom). (H) Expression of the common immune checkpoints. Red indicates high expression and blue indicates low expression. (I) The expression of some genes in the validation cohort. (J) The predicted proportion of C3 cluster cells in the validation cohort. (K) Number of DEGs between RespD376 and ARD614 in *CD8*+ T cells. (L) Venn diagrams represent 82 DEGs that were up-regulated in RespD376 and down-regulated in ARD614. (M) Functional enrichment modules of 82 DEGs, colored by module type.

Single-cell transcriptome data of T cells were extracted for re-clustering, and four subpopulations with transcriptional signatures were resolved (Fig 3A). First, the high expression of *GAPDH*, which indicated that glycolysis and exhaustion overexpressed in cell cluster 3 attracted our attention (Fig 3B). Further, investigation confirmed that the exhaustion-related markers *LAG3*, *SRGN* and *CD27* were all upregulated in cluster 3 (Fig 3C). Besides, both *CD8A* and *CD3E* were highly expressed, so cluster 3 was identified as an exhausted *CD8*+ T cell. Cytotoxic and proliferative phenotypes were also identified. For instance, the cytotoxicity-associated markers *GNLY*, *CSTW* and *CST7* were up-regulated in cluster 2, and cluster 3 expressed proliferation markers *MKI67* and *TYMS* counterintuitively (Fig 3D). Transcript levels of exhaustion marker *GAPDH* showed a positive correlation with *MKI67* for cluster 3 cells (Fig 3E). That may reflect the proliferative hierarchy for exhausted T cells maintenance in the context of chronic infections and cancer [24]. Quantitative analysis was performed to verify the existence of exhausted cells and elevated exhaustion score were observed for T cell cluster 3 (Fig 3F). Meanwhile, the developmental trajectory of the four clusters presented a binary branched structure (Fig 3G), cluster 2 cells as the root, and exhausted *CD8*+ T cells as the end state, which was consistent with previous studies [25,26]. In this process, T cell exhaustion-related immune checkpoints (*LAG3*, *CTLA-4*, *PD-1* and *TIGIT*) tended to be up-regulated, while synergist checkpoints (*KLRG1*) tended to be downregulated (Fig 3H). In addition, expressions of *LAG3*, *TIGIT* and proliferation markers *TYMS* and *MKI67* in MCC were also found to be higher than those in normal samples in the validation cohort (Fig 3I). Tumor immune invasion analysis also showed an increased estimated proportion of this cluster (C3) cells in MCC patients (Fig 3J). The cells in cluster 3 were exhausted *CD8* + T cell subpopulation in a state of proliferation, and T cell phenotypic composition evolved with MCC severity.

Subsequently, genes expression was compared among various time points. The difference analysis revealed 125 downregulated genes in ARD614 compared with pre-treatment, and 99 upregulated genes in RespD376 compared with ARD614 (Fig 3K). A total of 82 genes were strongly associated with disease progression after intersecting these genes (Fig 3L). The 82 genes mainly focused on cellular response to interferon−gamma, regulation of lymphocyte proliferation, and MHC protein complex binding (Fig 3M). Interferons (IFNs) consisted of different types such as class I IFN (α, β, ε, κ, ν, ω, τ, δ, ζ), class II IFN (γ), and class III IFN (λ). IFNs not only initiated anti-viral but also anti-tumor responses. In addition, class I and II IFNs caused an up-regulation of MHC molecules on the surface of the tumor cells [14,27].

### B cell and plasmablast changes across the MCC disease trajectory

**Fig 4:**
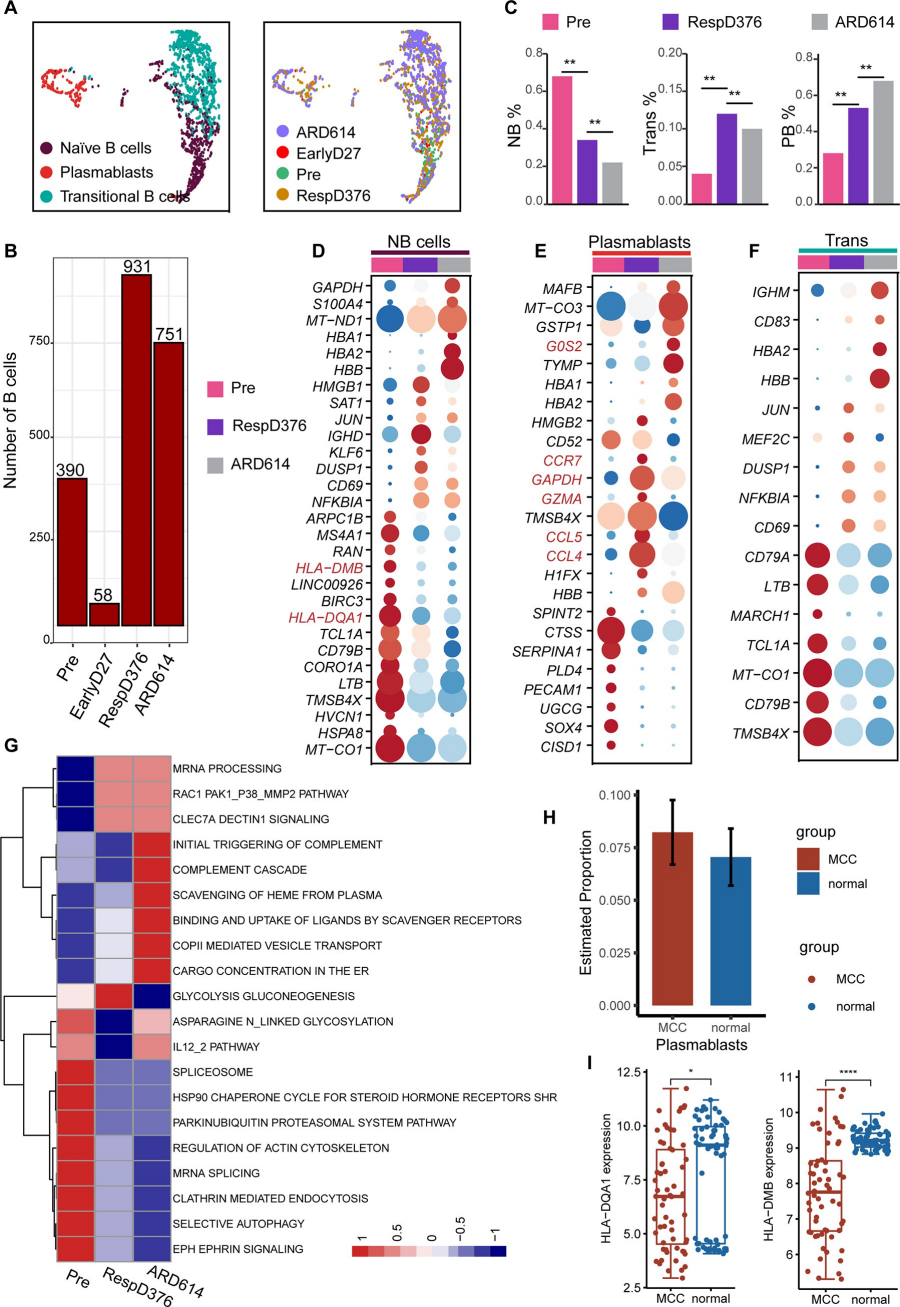
Elevated plasmablast levels as a feature of MCC. (A) The UMAP projection of 3434 B cells, colored according to B cell subtype (right) and time point (left). (B) The number of B cells at each time point. (C) The proportion changes of three B cell subtypes at each time point. (D-F) Dot plots for disease trajectory signature genes in naive B cells (NB), transitional B cells (Trans), and plasmablasts (PB). Genes were selected on the basis of the expression amount of the most characteristic genes. Color discriminates genes with upregulation (red) or downregulation (blue) expression, and point size represents the number of cells per group expressing the corresponding gene. (G) Metabolic pathways enriched in B cell subtypes. Top 20 active metabolic pathways in PBs are shown. Significant differences in metabolic activity were determined by using a Kruskal-Wallis test. (H) Proportion change of plasma cells. (I) Expression of HLA class II genes in the validation cohort.

We first extracted 3434 cells identified as B-cell lineages from integrated scRNA-seq data. UMAP package identified three distinct large clusters, including 778 naive B cells (NB), 210 transitional B cells (Trans), and 1142 plasmablasts (PB, Fig 4A). Since there were only 58 B cells at the stage of EarlyD27 and the cells at this stage were not of great significance for us to investigate the relapse mechanism, these 58 cells were excluded in the subsequent analysis (Fig 4B**)**. We observed a significantly decreased abundance of NB (up-regulation of *MS4A1* and *IGHD*) and increased abundance of PB (up-regulation of *CD38*) in ARD614 compared with Pre and RespD376 (Fig 4C).

We next explored the longitudinal gene expression patterns of NBs, PBs and Trans (Fig 4D–4F). The expression level of MHC class II genes (*HLA-DQA1*, *HLA-DMB*) in NB was associated with disease progression. The expression of MHC II genes was highest at the time point of Pre and significantly down-regulated at the ARD614 (Fig 4D), which suggested a disorder of immune crosstalk between adaptive immune cells and a decline in B cell antigen presentation. The chemokine receptors (*CCL5*, *CCL4*, and *CCR7*) were present in the PBs at the stage of RespD376, yet such genes were absent from PBs in patients with recurrence (ARD614) (Fig 4E). This could inhibit germinal center (GC) reactions and ultimately lead to the dysregulated persistent immune responses in the later stage of immunotherapy [28]. The expression of the activator protein-1 complex member (*JUN*) was upregulated in the Trans at RespD376, which may indicate that the tissue was primed for malignant proliferation before the appearance of recurrence features (Fig 4F). The expression of *G0S2* which was a potential regulator of metabolic changes increased at the stage of ARD614 [29]. Thus, metabolic pathway activities were assessed using a dataset of 85 metabolic pathways described in previous studies [23] (Fig 4G). There was a significant increase in glycolysis and gluconeogenesis in RespD376. Although Resp376 is a phase of recovery for MCC patients, it may indicate a delayed response between activation of drug metabolism and acquisition of drug resistance. PBs from the RespD376 state displayed a high metabolic activity, which was reduced only upon the ARD614 stage. Many metabolic processes in PBs increased: including propanoate and pyruvate metabolism, arachidonic acid metabolism, steroid biosynthesis, glycolysis and gluconeogenesis. PBs can act as a nutrient sink that regulates the immune response [30]. Metabolic hyperactivity of PBs in ARD614 may result in nutrient deprivation of the GC reaction which will hinder the generation of long-lived plasma cell and memory B cell responses [30]. In bulk RNA data, the abundance of PBs was found to be higher in MCC samples compared to normal samples (Fig 4H). Similarly, the NB marker *HLA-DQA1* was upregulated in normal tissues compared to MCC (Fig 4I). Altogether, the analysis identified the widespread activation of PBs and suggested that increased drug metabolism and inhibition of GC reaction by the rapid development of PBs may be one of the main mechanisms of drug resistance and relapse.

### *S100A*^high^*HLA-DR/DP*^low^ monocyte subpopulation reflects coordinated changes with both the immunotherapy progression and MCC severity

**Fig 5:**
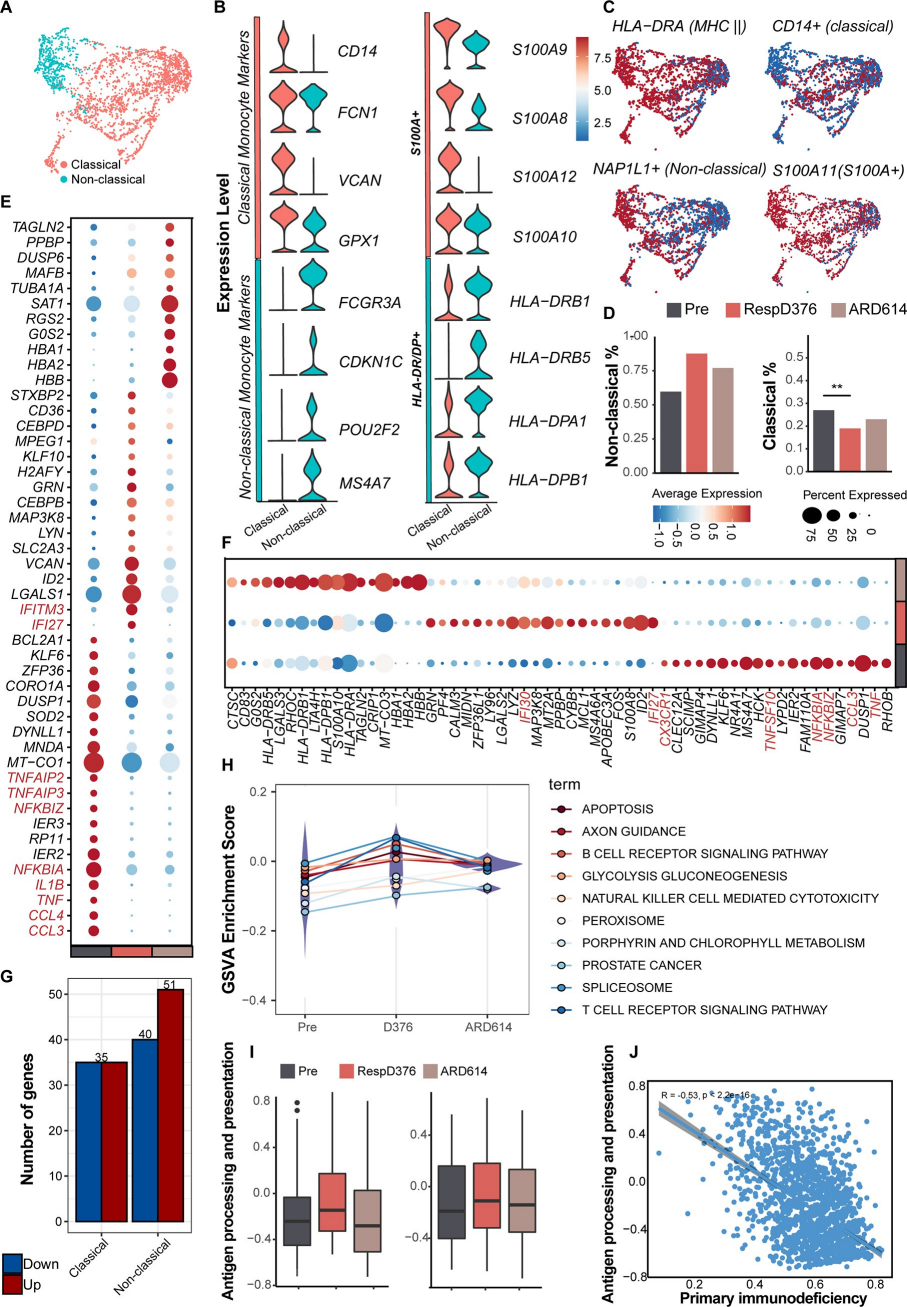
Monocyte subpopulation reflects coordinated changes with both the immunotherapy progression and MCC severity. (A) UMAP embedding of all monocytes colored by monocyte compartment subtype. (B) Violin plots showing the expression of non-classical and classical monocytes, *S100A*, *HLA-DR/DP* markers in monocyte subtypes. (C) Several cell phenotypic markers were used to identify monocyte cell subtypes. (D) The proportion changes of two monocyte subtypes at each time point. (E) Dot plot for disease trajectory signature genes in classical monocytes. Color discriminates genes with upregulation (red) or downregulation (blue) expression, and point size represents the number of cells per group expressing the corresponding gene. (F) Dot plot for disease trajectory signature genes in non-classical monocytes. (G) Number of DEGs between RespD376 and ARD614 in two monocyte subtypes. (H) The top 10 GSVA results of non-classical monocytes. Y-axis represents the expression level of the entire pathway. (I) Changes in levels of “antigen processing and presentation pathways” in two monocyte subtypes. (J) Correlation between pathway “primary immunodeficiency” and pathway “antigen processing and presentation” in monocytes.

UMAP visualization of all 2489 monocytes showed significant separation of non-classical and classical monocytes (Fig 5A). Classical monocytes were characterized by the upregulated transcripts *CD14*, *FCN1*, *VCAN*, and *GPX1*, while non-classical monocytes were identified based on the up-regulation of *FCGR3A*, *CDKN1C*, *POU2F2*, and *MS4A7* (Fig 5B). The classical monocyte cluster exhibited expression of inflammation-related transcripts such as *S100A8*, *S100A9*, *S100A10*, and *S100A12*, and decreased levels of MHC II transcripts such as *HLA-DR/DP* (Fig 5B and 5C). The classical monocyte (*S100A*^high^*HLA-DR/DP*^low^) fraction increased in Pre and ARD614, compared to the RespD376. The non-classical monocyte cluster (*S100A*^low^*HLA-DR/DP*^high^) percentage increased for RespD376 stage (Fig 5D). Interestingly, the characteristic gene expression patterns of the two cell subtypes in the protein family (Type I IFN response genes *IFITM3* and *IFI27* in the classical cluster, *IFI27* and *IFI30* in the non-classical cluster, for instance) were both highly expressed in RespD376, yet these genes were absent from monocytes in ARD614. Meanwhile, general down-regulation of the pro-inflammatory factors such as TNF family (*TNF*, *TNFAIP2*, *TNFAIP3* and *TNFSF10*) and chemokine family (*CCL3*, *CCL4* and *CX3CR1*) were present in RespD376 until disease relapse (ARD614) (Fig 5E and 5F). Differential gene analysis of these two cell types revealed that the classical monocyte had 35 genes upregulated and 35 genes downregulated, while the non-classical monocyte had 51 genes upregulated and 40 genes downregulated in expression (Fig 5G). The proportion changes and transcript characteristics of the two monocyte subtypes at different periods seemed to suggest that the improvement of MCC benefited from a peripheral environment with low inflammation and high immune response, prompting us to explore this conjecture more.

The GSVA results of non-classical monocytes (*S100A*^low^*HLA-DR/DP*^high^) showed that apoptosis, B cell receptor signaling pathway, glycolysis gluconeogenesis, natural killer cell mediated cytotoxicity and T cell receptor signaling pathway were more active during convalescence (pseudotime RespD376) (Fig 5H). The corresponding biological significance of HLA class II genes expression signatures were shown in Fig 5I. In both classical and non-classical monocytes, the ability of antigen processing and presentation in convalescence was stronger than those in the pre-treatment and relapse. Furthermore, antigen processing and presentation was negatively correlated with the primary immunodeficiency (Fig 5J), suggesting that normal functioning of monocyte after treatment may significantly reduce tumor pressure until recurrence.

## Discussion

A comprehensive understanding of the various cellular changes in MCC patients is essential to determine the effectiveness of treatment, predict disease prognosis, and understand the heterogeneity of reported disease severity. In this report, we mainly discussed the following aspects:

The significant difference observed between the response phase and the relapse phase in peripheral immune cells is that exhausted *CD8*+ T cells (T cells that gradually lose their function) [31] were significantly elevated and exhibited abnormal phenotypes. Previous studies found that increased infiltration of *CD8*+ T cells effectively improve the prognosis of patients with MCC [32]. The increase of *CD8*+ T cells was also observed in the response period (RespD376), which seemed to explain the decrease in tumor pressure in MCC patients during the RespD376. We also identified a cluster of *CD8*+ T cells that amplified during the RespD376 and showed both exhaustion and proliferation phenotypes. This was consistent with the previous finding that infiltrating T cells were usually characterized by an exhausted phenotype [33,34]. The impact of exhausted phenotype T cell effectors on patients may be greater than the activity of their CD8+ T cells expanded in vitro, leading to the gradual depletion of these T cells used for immunotherapy and loss of their therapeutic efficacy. Meanwhile, the expression of immune checkpoints *LAG3*, *CTLA-4*, *PD-1*, and *TIGIT* in this cluster was higher than those in other clusters. Both exhaustion of cells and upregulation of these immune checkpoints inhibit the immune response and can be used by tumor cells as an immune escape mechanism, which further led to the failure of immunotherapy [35]. In addition, the results revealed that T cell infiltration level was not an important independent predictor of survival in MCC patients.

The results of our longitudinal study identified two other cellular features associated with MCC recurrence that previous studies had not recognized. First, the relative abundance of PBs with low expression of chemokine receptors (*CCL5*, *CCL4*, and *CCR7*) and high metabolic activity increased during the relapse phase (ARD614). Dynamic changes in chemokine receptor expression induce antigen-stimulated B cells to target specific lymphoid tissue sub-compartments, resulting in further activation and proliferation of B cells [36]. During the immune process, B cells upregulate the receptor for *CCL19/21* (*CCR7*), produced in the paracortex, causing them to migrate to the T cell zone. Activated B cells also secrete T chemokines *CCL3* and *CCL4*, thus increasing the efficiency of interaction with activated T cells that also migrate to the T:B boundary [37]. So, high expression of chemokines not only activate B cells to produce an effective immune response but also improve the synergistic efficiency of T cells and B cells. At the same time, both the dysregulation of these chemokines and the metabolic hyperactivity of PBs in the ARD614 will hinder the formation and sustainment of germinal centers, which will lead to the interruption of B cell-mediated long-lasting and broadly protective antiviral immune response, further compromising the effectiveness of immunotherapy [30,38].

A significant increase of high inflammatory classical monocytes was identified, which carried a weak MHC II signature. Chronic inflammatory diseases such as rheumatoid arthritis was associated with a higher incidence of MCC [39]. There was also a significant correlation between inflammatory infiltration and *PD-L1* expression in tumor cells [40]. Hence, we speculated that the upregulation of pro-inflammatory factors and inflammatory transcripts at the ARD614 may create a local pro-inflammatory environment that drives the expression of tumor *PD-L1* [41]. Meanwhile, extensive downregulation of MHC II was observed in T cells, B cells, and monocytes during the ARD614, which confirmed the importance of immunity as a prognostic determinant of MCC patients. These results clearly suggested that regulatory events in T cells, B cells, and monocytes might act as pivotal components of an unfavorable course of MCC, which mandates further prospective exploration.

There are several limitations of our study. First, the work contains no wet lab cross-check. Second, with the current scRNA-seq strategy, peripheral blood samples are not a complete representation of the true state of MCC. Therefore, future studies with new scRNA-seq data and validation in a larger clinical cohort may help to further investigate the mechanisms of immunotherapy tolerance in MCC.

## Conclusions

In this study, computational methods were used to integrate single-cell characteristics at different time points to form an integrated and comprehensive view of MCC that relapsed after immunotherapy. A series of adverse factors that may lead to immune escape in MCC were determined, including T cell exhaustion and immune crosstalk disorders. The peripheral environment with high inflammation and low immune response may also lead to resistance to immunotherapy.
